# Probability Distribution and Recommended Value for the Crack Spacing Reduction Coefficient of Steel Fiber in SFRC Beams

**DOI:** 10.3390/ma19091704

**Published:** 2026-04-23

**Authors:** Yunchao Huang, Jiachen Sun, Danying Gao, Shangke Li, Changhui Zhang, Huanhuan Yan, Kangbo Qiao

**Affiliations:** 1 School of Water Conservancy, North China University of Water Resources and Electric Power, Zhengzhou 450046, China; hyc1308360205@126.com (Y.H.); sjc18240883657@126.com (J.S.); lishangke@ncwu.edu.cn (S.L.); zhangchanghui@ncwu.edu.cn (C.Z.); yanhuanhuan@ncwu.edu.cn (H.Y.); 15237855053@163.com (K.Q.); 2 School of Water Conservancy and Transportation, Zhengzhou University, Zhengzhou 450002, China

**Keywords:** steel fiber reinforced concrete, crack width, reduction coefficient, statistical analysis, guarantee rate

## Abstract

The effect of steel fiber on the maximum crack width of steel fiber reinforced concrete (SFRC) members under normal service conditions was studied through tests on a series of 19 flexural beams, and the applicability of the crack spacing reduction coefficient of steel fiber in SFRC beams from Modified Rilem model that using for calculating maximum crack width was discussed, of which the reduction coefficient value was used to determine the influence of steel fibers on the crack width. Results show that the maximum crack width of the RC beam under bending conditions clearly decreased with the addition of fibers, and the reduction coefficient value of the crack width of the SFRC beam is greater when compared with that specified in the Modified Rilem model. The effect of steel fiber on reducing crack width was overrated, while the reduction coefficient proposed in the Modified Rilem model was used to calculate the maximum crack width. The probability of the reduction coefficient was analyzed based on experimental data collected from the relevant literature, and the reduction coefficients for different steel fiber types with various guarantee rates were obtained. Finally, the suggestion for the value of the reduction coefficient in the Modified Rilem model was proposed to be 1.19, which could be referred to for the code revision.

## 1. Introduction

With the development of concrete structures in terms of high crack resistance, high durability, and high bearing capacity, as well as the in-depth application in special service conditions and harsh environments, concrete materials are continuously advancing to achieve high strength and ductility. Steel fiber reinforced concrete (SFRC) is a composite material created by incorporating an appropriate quantity of steel fibers into ordinary concrete. The randomly oriented and discontinuous steel fibers are wrapped in the cement mortar of concrete and have a reliable adhesion, resulting in fibers that can effectively reduce the rate of load drop after peak load, and help to prevent the crack propagation owing to the transference of tension stresses across the cracked section. The use of high-strength steel bars in building engineering has become an unavoidable trend in the face of increased performance demands, as well as issues pertaining to cost-effectiveness and environmental preservation. To fully utilize the tensile properties of high-strength steel bars, they need to be used synergistically with SFRC, and particularly high-strength SFRC. This combination enables the full utilization of the inherent advantages for both materials, endowing cracked concrete with dependable post-cracking tensile strength and ductility.

Concrete cracking resistance is an important factor for underpinning both structural performance and durability, and it was equally crucial, aside from the necessity of fulfilling the load-bearing capacity criteria for concrete structures. Adding steel fibers to reinforced concrete (RC) beams can change the failure modes [1], increase load-bearing capacity [2] and ductility [3], and decrease crack width and deflection [2] as has been shown in previous studies. There is an average increment of about 35% for the cracking moment of RC beam with steel fibers (SFRC beam) when compared with ordinary RC beams, which is due to the crack resistance capacity afforded by steel fiber [3]. The crack width calculation methods for SFRC and RC beams were different in existing codes: GB 50010-2010 in China [4], Eurocode 2 [5], and ACI 318 [6] are generally applicable to RC beams, while the effects of steel fiber are considered in the RILEM TC-162-TDF [7] and JGJ/T 465-2019 [8]. The maximum crack width calculation model was investigated according to RILEM TC 162-TDF [7] by Touhami Tahenni [9], and a Modified Rilem model [9] was proposed with a reduction coefficient of the crack spacing k, taking the quantity of fibers, fiber length, aspect ratio, and fiber orientation into consideration. Nevertheless, there is no reduction coefficient reference value that corresponds to various types of steel fiber in the model; only a reference value of 0.8 is recommended. When the reduction coefficient of crack spacing k is used to calculate the maximum crack width of a beam under bending, this can be used to determine the effect of steel fiber. The majority of studies conduct regression analyses on the test results [9,10,11,12]. The appropriate value of the reduction coefficient can be determined based on the mean of the regression value [13] or combined with reliability theory analysis [14]. In particular, the specified value for RC beams in the code was directly applied to SFRC beams in some studies [15].

However, there are currently no studies that derive the reduction coefficient according to the existing calculation model for the maximum crack width of beams in the code, integrating a large quantity of experimental data collected from relevant studies, and then performing a statistical analysis on the impact of reduction coefficient, ultimately making some suggestions for revising the specified value of reduction coefficient in the code according to the results. Moreover, many different fiber types have emerged recently and have been used in RC beams; it is therefore uncertain as to whether the reduction coefficient in the model is suitable for RC beams employing these new types of steel fibers. Consequently, research into the reduction coefficient for SFRC beams must be carried out, and a suitable guarantee rate should be determined to ensure that the current model is able to satisfy all the requirements of structural applications [15,16].

Flexural tests were performed on 19 beams in this paper. Based on the experimental results of a total of 30 sets of SFRC beams, including both the data generated in the current study and data collected from other relevant studies, the reduction coefficient of crack spacing for flexural beams was calculated using the model from RILEM TC-162-TDF [7,9]. In addition, the values of the reduction coefficient for various types of steel fiber were determined according to the results of statistical analysis, considering varying guarantee rates. Finally, a suggestion for revising the value of the reduction coefficient in the Modified Rilem model [9] was proposed.

## 2. Experimental Program

### 2.1. Materials

**Figure 1:**
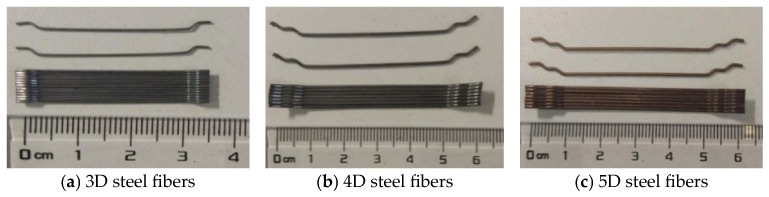
Types of steel fibers. Reprinted from reference [1] with permission from Elsevier.

The concrete in the test was designed to be formulated with strength grades of C40, C60, and C80, where C60 indicates that the cubic compressive strength of concrete is no less than 60 MPa. Ordinary Portland cement of 42.5 grade that met the requirements of GB 175-2023 [17] in China was utilized, along with clean tap water. The aggregates comprised natural river sand (fineness modulus 2.84) and limestone gravel (particle size 5–20 mm). To promote fiber dispersion uniformity and enhance workability, a liquid polycarboxylate water-reducing admixture was applied. Three types of steel fiber produced by Bekaert Building Products Hong Kong Ltd. (Shanghai, China) were used, all featuring an aspect ratio of 65, as depicted in Figure 1.

The longitudinal reinforcements in the beams consisted of steel bars with varying nominal diameters, and the properties of these bars were measured by subjecting them to tensile strength tests. The physical and mechanical characteristics of the steel bars are summarized in Table 1. The HRB500 indicates the yield strength of the steel bar is greater than 500 MPa.

### 2.2. Specimen Design and Preparation

Flexural properties were studied by testing 19 rectangular beams to failure under a four-point loading scheme. The RC beams were designed with a 150 mm × 300 mm cross-sectional area and a 3000 mm total length, and the shear span length and the distance between two loading points in the constant moment zone were both set at 900 mm.

The parameters and actual sizes of the tested beams are detailed in Table 2. The longitudinal tension reinforcement was provided by two to four deformed steel bars, whereas two 10 mm steel bars were used at the compression sides of all specimens to hold the shear stirrups in place. Two-legged stirrups with a 10 mm nominal diameter at 100 mm spacing within the shear span zone were used to provide adequate resistance against shear failure, and a concrete cover of 20 mm to the transverse reinforcement.

The mixture proportions and mixing process of the concrete were the same as those in the literature [16]. Wooden molds were employed for casting the beam specimens, with meticulous mixing procedures being implemented to improve the uniform distribution of steel fibers. To test the concrete performance related to each beam specimen, six concrete cubes and prismatic specimens were cast using plastic molds to determine the basic mechanical properties of concrete according to GB/T 50081-2019 [18]. These specimens were cast from the same concrete batch mixture as beam specimens, cured under the same conditions, and tested at the same time as beam specimens. The average strength of each concrete mixture was derived from testing three cubic and prismatic specimens.

### 2.3. Test Setup and Procedure

**Figure 2:**
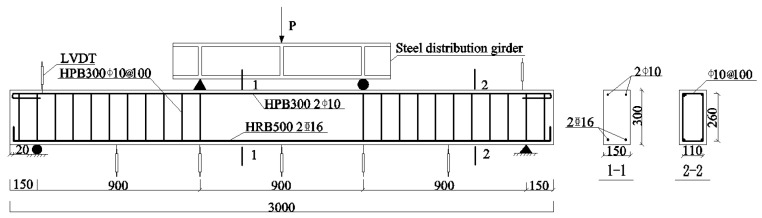
Loading and measuring arrangements (units: mm).

The specimens were loaded and measured using the arrangement depicted in Figure 2. A 2000 kN hydraulic servo testing machine provides by Shanghai Hualong Test Instruments Corporation (Shanghai, China) was employed to apply monotonic loading under a four-point bending arrangement, and the equal loads were imposed at the loading points, each positioned at one-third of the span length from the supports. A universal testing machine (UTM) with a load capacity of 3000 kN was employed to conduct tests on the concrete cubes and prisms.

To ensure full contact, the specimen was subjected to a preload of 10 kN, followed by unloading to 0 kN. The load was applied step by step until failure during the formal loading process and held for 10 min at each load level to determine whether the cracks were produced and extended. The load was increased monotonically in steps of 5 kN before cracking, and an increment of 10 kN was applied after cracking until 85% of the ultimate load was reached, while the development trends of cracks were observed and drawn along with the corresponding load for each increment of 20 kN. The deflections were continuously measured with the help of linear variable displacement transducers (LVDTs) with a measurement range of 100 mm. The crack width at the location parallel to the axis of longitudinal bars was measured and recorded with each load increase of 20 kN, and the largest crack width value within the purely bending section of the beam was taken as the maximum crack width under the corresponding load level. The test was conducted under displacement control at a rate of 0.5 mm/min and maintained to failure, and a real-time data-acquisition system was used to monitor and record load–displacement response.

## 3. Analysis of Experimental Results

### 3.1. Appearance and Development of Cracks

**Figure 3:**
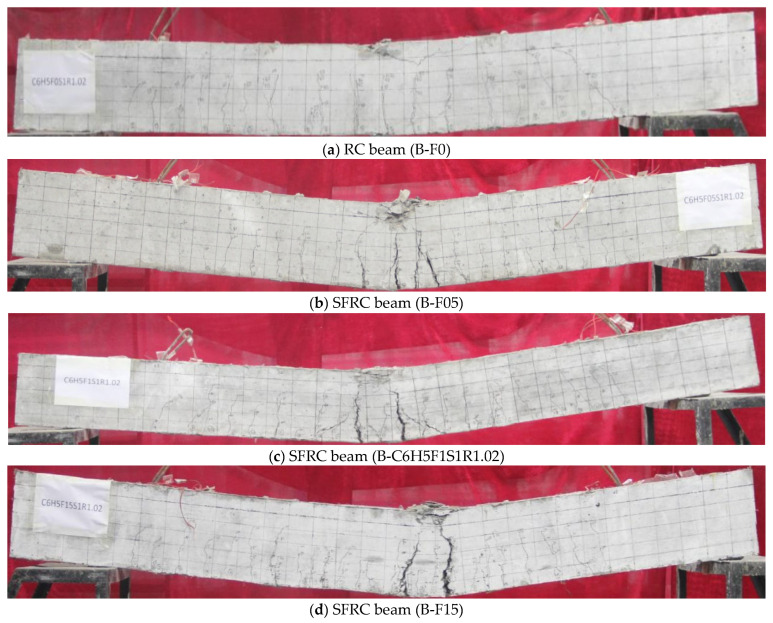
Typical failure modes and crack patterns. Reprinted from reference [1] with permission from Elsevier.

All beam specimens demonstrated bending failure. Typical crack patterns are shown in Figure 3. The damaged section is located in the pure bending zone, which is approximately 300 mm around the midspan section. The first crack usually occurs in the tension zone of the pure bending section. The load level at the first crack is 30 kN for RC beams, the crack height is about 100 mm, and the width is 0.03 mm. The corresponding loads for the beam specimens with various steel fiber volume fractions are 20 kN, 30 kN, and 30 kN, respectively; the initial crack height is about 60 mm, 60 mm, and 15 mm, respectively, and the width is about 0.02 mm. The results indicate that longer steel fibers have little effect on cracking moments, as the 3D steel fiber with a length of 35 mm was used in the test beam. However, it can effectively limit the initial crack width, and noticeably smaller crack heights are found for the SFRC beams. Although the unevenness of concrete increases with the addition of steel fibers, particularly in the crack width measurement position that is parallel to the axis of tensile longitudinal reinforcement, where concrete is not amenable to compact vibration, and steel fiber is difficult to uniformly disperse, this does not affect the effectiveness of steel fiber in preventing and limiting cracks after crack propagation.

The maximum crack width of RC beam specimens is 0.14 mm when the load is 60 kN, and the maximum crack widths of SFRC beam specimens are 0.12 mm, 0.1 mm, and 0.08 mm under the same load. The crack width of beams is observed to diminish more significantly with higher steel fiber volume fractions, and the maximum crack width of the SFRC beam specimen with 1.5% steel fiber volume fraction reduces by more than 40% relative to the RC beam specimen. The load released by cracked concrete at the crack section is partially supported by steel fibers crossing the crack. Owing to the fiber-bridging effect, the steel fibers can bear tensile force together with reinforcement, which results in reduced stress applied to reinforcement at the crack section, and the increasing speed of reinforcement strain slows down. Furthermore, the fiber-bridging action facilitates stress transmission and dispersion over a broader area, effectively mitigating stress concentration at crack tips, also increasing the mechanical occlusion force and reducing bond slip between steel bar and concrete [19], thus effectively reducing the crack width of RC beams. When the load exceeds 60% of ultimate load for beam specimen when the load is 100 kN, and this is less than the yield load, there are basically no new cracks appearing in pure bending section, the number of cracks within the 900 mm length in pure bending zone of RC beam is 8, while cracks in the pure bending section of the SFRC beam number at 10, 13, and 15, respectively. The reduction in crack spacing under the same load is obvious due to the fact that steel fibers shorten the stress transfer distance required for secondary cracks [20], since the tensile stress is jointly resisted by steel fibers and steel bars at the crack section, well distributed into the surrounding concrete, and frequently exceeding the concrete tensile strength [21]. In short, the addition of steel fiber led to an increase in the number of cracks, and reduced the crack spacing and width, as the fibers spanning the cracked section of concrete inhibit the propagation and widening of cracks.

The number of cracks no longer continues to increase after the steel bar yields, and the crack spacing tends to stabilize. The deflection and crack width of the beam increase quickly, the crack height extends to the compression zone of the beam while the load increases very slowly, and a portion of the secondary cracks near the main cracks are gradually converging to it. The concrete in the compression zone forms the horizontal cracks and is gradually crushed after the yielding of tensile reinforcement for the RC beam. The beam experiences a sudden brittle failure under a small deflection. For the beam specimens with steel fiber in the compression zone, one or two of the cracks in the pure bending zone exhibit significant widening after the yielding of the reinforcement. The steel fiber increased the ductility of SFRC beams considerably and delayed the final concrete crushing. The ultimate deformation capacity of concrete in the compression zone of SFRC beam specimens is improved by the incorporation of steel fibers, resulting in ductile and smooth concrete compression failure.

### 3.2. Prediction Models of the Crack Width

The crack width of the SFRC beam can be predicted via several theoretical models that are presented in the existing design specifications. The following methods can be used to calculate the crack width of tested beams.

#### 3.2.1. Rilem TC 162 Model

The model for predicting crack width in RC beams containing steel fiber, as presented in Rilem TC 162-TDF code [7], is given below:

(1) $$w_{R}=s_{rm}\varepsilon_{sm}$$

where $w_{R}$ is the maximum crack width; $s_{rm}$ is average crack spacing (mm), given by the following:

(2) $$s_{rm}=(50+0.25k_{1}k_{2}\frac{\varphi }{\rho_{r}})\frac{50}{l_{f}/d_{f}}$$

where $k_{1}$ is taken as 0.8 for high yield bars, and for mild steel bars, it is 1.6; $k_{2}$ is taken as 0.5 for flexure; φ is the reinforcing bar diameter (mm); $\rho_{r}$ is the effective steel percentage (%); $l_{f}$ is the fiber length (mm); $d_{f}$ is the fiber diameter (mm).

The average strain in tension $\varepsilon_{sm}$ can be described by the following equation:

(3) $$\varepsilon_{sm}=\frac{\sigma_{s}}{E_{s}}\left[1-\beta_{1}\beta_{2}{\left(\frac{\sigma_{sr}}{\sigma_{s}}\right)}^{2}\right]$$

where $\sigma_{s}$ is tension stress of reinforcing bars (MPa); $E_{s}$ is the modulus of elasticity of the steel bar (MPa); $\beta_{1}$ is taken as 1.0 for high-yield deformed bars, where for mild steel bars, it is 0.5; $\beta_{2}$ is a coefficient related to loading duration, which is 1.0 for short-term loading, where for long-term loading, it is 0.5; and $\sigma_{sr}$ is stress in the tension reinforcement, and the value is derived from an uncracked concrete section under loading conditions that result in initial cracking (MPa).

A Modified Rilem model [9] was proposed on the basis of RILEM TC 162-TDF [7], which contains a reduction coefficient of crack spacing applied to crack width, which is related to the quantity of fiber, aspect ratio, and orientation of fibers. The calculation model is proposed as follows:

(4) $$w_{MR}=\left(50+0.25k_{1}k_{2}\frac{\varphi }{\rho_{r}}\right)\frac{50k}{\frac{l_{f}}{d_{f}}+\alpha V_{f}}\frac{\sigma_{s}}{E_{s}}\left[1-\beta_{1}\beta_{2}{\left(\frac{\sigma_{sr}}{\sigma_{s}}\right)}^{2}\right]$$

where $w_{MR}$ is the maximum crack width obtained by the Modified Rilem model; k is the reduction coefficient of crack spacing related to fiber, which is taken as 0.8; $l_{f}/d_{f}$ is aspect ratio; $V_{f}$ is the percentage by volume of fiber; α is the fiber orientation factor, which is calculated from the model proposed by Soroushian [22], expressed as follows:

(5) $$\alpha =0.31\frac{l_{f}}{b}+0.64$$

where b is the beam section width (mm).

#### 3.2.2. Eurocode 2 EN1992-1-1 Model

According to the Eurocode 2 (EN1992-1-1) code [5], the width of a crack can be determined by the following equations.

(6) $$w_{E}=s_{rm}\left(\varepsilon_{sm}-\varepsilon_{cm}\right)$$

where $w_{E}$ is maximum crack width; $s_{rm}$ is average crack spacing (mm). For section under flexure conditions, it can be calculated according to the following formula:

(7) $$s_{rm}=3.4C+0.425k_{1}k_{2}\varphi /\rho_{eff}$$

where C is concrete cover (mm); $k_{1}$ is a bond coefficient, taken as 0.8 for high yield bars, and where for mild steel bars, it is 1.6; $k_{2}$ is equal to 0.5 in flexure; φ is diameter of reinforcing bar; and $\rho_{eff}$ is effective steel percentage, taken as $A_{s}/A_{ceff}$, where $A_{s}$ is the reinforcement area, and $A_{ceff}$ is the effective concrete area.

Where $\varepsilon_{sm}-\varepsilon_{cm}$ is average tension strain, in which $\varepsilon_{sm}$ is average tension strain of steel bar (mm), and $\varepsilon_{cm}$ is average tension strain of concrete. It can be solved by the following equation:

(8) $$\varepsilon_{sm}-\varepsilon_{cm}=\frac{\sigma_{s}}{E_{s}}-\frac{k_{t}f_{ctm}\left(1+n\rho_{eff}\right)}{E_{s}\rho_{eff}}$$

where $\sigma_{s}$ is tension stress of reinforcing bars (MPa); $E_{s}$ is elastic modulus of steel bar (MPa); $k_{t}$ is taken as 0.6 for short-term loading; $f_{ctm}$ is tension strength of concrete (MPa); and n is modular ratio $E_{s}/E_{c}$, where $E_{c}$ is the elastic modulus of concrete.

#### 3.2.3. ACI 318 Model

The crack width model for bending beams presented in the American design code ACI Committee 318 (2019) [6] was proposed by Gergely P [6,9,23,24] (ACI 318 (Gergely & Lutz)). It is presented below:

(9) $$w_{AG}=0.076\beta f_{s}\sqrt[{3}]{d_{c}A}$$

where $w_{AG}$ is the maximum crack width predicted by the calculation method proposed by Gergely P; β can be taken as 1.2, which is the ratio of the distance from the neutral axis to the tension face to that from the centroid of reinforcement to the tension face; $f_{s}$ is the tension stress of reinforcement (ksi), which can be taken as $f_{s}=0.6f_{y}$, where $f_{y}$ is yield strength; $d_{c}$ represents concrete cover (in); and A is the tension section area of concrete, which is calculated as the total effective tension area of concrete surrounding the bars (with a shared centroid) divided by the number of bars (in^2^).

The formula suggested by Frosch [6,10,24,25] (ACI 318 (Frosch)) is expressed as follows:

(10) $$w_{AP}=2000\frac{f_{s}}{E_{s}}\beta \sqrt{d_{c}^{2}+{\left(\frac{s}{2}\right)}^{2}}$$

where $w_{AP}$ is the maximum crack width obtained by the calculation model established by Frosch; $E_{s}$ is the elastic modulus of reinforcement (ksi); $d_{c}$ is the distance from the tensile surface to the center of reinforcement, which is closest to the surface (in); s is the maximum reinforcement spacing (in).

According to the ACI Committee 318 (2019) [6], the formula for rebar spacing is as follows:

(11) $$s=15\left(\frac{40000}{f_{s}}\right)-2.5c_{c}\leq 12\left(\frac{40000}{f_{s}}\right)$$

where $f_{s}$ is the calculated stress in reinforcement (psi).

#### 3.2.4. JGJ/T 465-2019 Model

The formula to calculate the maximum crack width of SFRC beams with a rectangular section, incorporating the effects of steel fiber, which was suggested by the Technical Specification for Fiber Concrete Structures (JGJ/T 465-2019) in China [8], is given below:

(12) $$w_{f\max}=w_{\max}\left(1-\beta_{cw}\lambda_{f}\right)$$

where $w_{f\max}$ is the maximum crack width of SFRC beams (mm); $w_{\max}$ is the maximum crack width of RC beams (mm); $\beta_{cw}$ is the impact coefficient of the crack width of fiber, which can be taken as 0.35 for the bending members; $\lambda_{f}$ is the characteristic value of fiber, which can be calculated by the following equation: $\lambda_{f}=V_{f}l_{f}/d_{f}$, where $V_{f}$ is the percentage by volume of fiber, $l_{f}$ is the fiber length (mm), and $d_{f}$ is the fiber diameter (mm).

The maximum crack width $w_{\max}$ of RC beams with a rectangular section is calculated according to the model stipulated in the Code for the Design of Concrete Structures (GB 50010-2010) in China [4]. The equation is as follows:

(13) $$w_{\max}=\alpha_{cr}\psi_{s}\frac{\sigma_{s}}{E_{s}}\left(1.9c_{s}+0.08\frac{d_{eq}}{\rho_{te}}\right)$$

where $\alpha_{cr}$ is taken as 1.9 for reinforced concrete flexural members, which represents the characteristic force coefficient of the member; $\psi_{s}$ is the strain inhomogeneity coefficient of longitudinal reinforcement in the tension zone between cracks, which ranges from 0.2 to 1.0, and it can be calculated by the following formula: $\psi_{s}=1.1-0.65f_{tk}/\rho_{te}\sigma_{s}$, where $f_{tk}$ is the standard value of axial tensile strength of concrete (MPa), $\rho_{te}$ is longitudinal tensile reinforcement rate calculated by the effective tensile cross-sectional area of concrete, and $\sigma_{s}$ is the equivalent stress of longitudinal tensile reinforcement of a reinforced concrete member (MPa); $E_{s}$ is the elasticity modulus of the steel bar (MPa); $c_{s}$ is the thickness of the protective layer of concrete (mm); $d_{eq}$ is the equivalent diameter of the tensile steel bar (mm).

### 3.3. Comparison of Predicted Crack Widths with Measured Results

The comparison of the predicted value $w_{p}$ according to the codes mentioned above with the measured result $w_{t}$ of crack widths is presented in Table 3. For the crack widths of beam specimens predicted by Eurocode 2 [5], the ratios of predicted crack widths to experimental results vary from 0.9797 to 2.4950, with a mean value of 1.6091, and the value predicted by Eurocode 2 [5] is notably small. For the crack widths of beam specimens calculated by the ACI 318 (Gergely & Lutz) [6,9,23,24] model, the ratios of predicted results to the experimental crack widths are less than 0.67; the crack width predicted by the ACI 318 (Gergely & Lutz) [6,9,23,24] model is larger than that measured in the experiment. A larger predicted crack width was also obtained by ACI 318 (Frosch) [6,10,24,25], as the ratios of $w_{t}/w_{p}$ are less than 0.25. The predicted value is slightly larger as the ratios of $w_{t}/w_{p}$ with a mean value of 0.9154 according to GB50010-2010 [4], in which the crack widths predicted using the model were in good agreement with the experimental results. Furthermore, the influence of steel fiber has not been taken into account in Eurocode 2 [5], ACI 318 (Gergely & Lutz) [6,9,23,24], ACI 318 (Frosch) [6,10,24,25], and GB 50010-2010 [4].

The influence of fiber is taken into account when the model in JGJ/T 465-2019 [8] was used to predict the crack width of SFRC beam specimens, and the $w_{t}/w_{p}$ ratios with a mean value of 1.0865, which is closest to the experimental results. Four fundamental parameters of steel fiber are reflected in the model, namely, fiber volume fraction, fiber length, and fiber diameter, along with a reduction coefficient on the crack width of steel fiber. For the crack widths of beam specimens predicted by Rilem TC-162-TDF [7], where the $w_{t}/w_{p}$ ratios have a mean value of 1.2134; the predicted values are less than the measured results. Two fundamental parameters of steel fiber are reflected in this model, namely, fiber length and diameter. For the crack widths of beam specimens predicted by the Modified Rilem model [9], the ratios of $w_{t}/w_{p}$ vary from 1.0829 to 1.9964, with a mean value of 1.5169. Five fundamental parameters were taken into consideration in the Modified Rilem model [9], namely, fiber volume fraction, fiber length, fiber diameter, fiber orientation factor, and reduction coefficient of crack spacing k. The reduction coefficient was used to determine the influence of fibers in terms of decreased crack spacing.

Through the above comparison of predicted flexural crack widths of SFRC beams from multiple models with experimental results, it is evident that the influence of steel fiber should be considered in crack width prediction. More parameters are reflected in the Modified Rilem model [9], it is necessary to undertake an in-depth study of the model. A suitable reduction coefficient k that used to determine the influence of steel fiber must be proposed, as the mean value of $w_{t}/w_{p}$ is greater than one. The personal test is not representative due to the disunity and inaccuracy of the testing method, the deficiency of the test data, and the fact that the reduction coefficient of steel fiber has not been classified according to fiber type. Therefore, the Modified Rilem model [9] needs to be revised and improved, taking into account the experimental results collected from the relevant literature. The reduction coefficient k must be further studied to improve the agreement between model predictions and experimental results.

### 3.4. Calculation Method for Reduction Coefficient of Crack Spacing as Applied to Crack Width

There are currently three main theories for calculating crack width. These are the bond-slip theory, no-slip theory, and comprehensive theory. The crack spacing depends on the tensile strength of concrete, the ratio and diameter of reinforcement, and the average bond stress at the interface according to the bond-slip theory. The crack width is the strain difference between reinforcement and concrete within the range of crack spacing. The no-slip theory considers that crack width is mainly related to the thickness of concrete cover. Neither of them can fully explain the test phenomena and data. It is believed that bond-slip between the steel bar and concrete, as well as the thickness of concrete cover, are important factors affecting the crack width in the comprehensive theory [26]. The experimental study and theoretical analysis of SFRC show that crack width is effectively reduced with the addition of steel fiber, and the crack width decreases more obviously with the increase in volume fraction [21]. The bond-slip and cover thickness of SFRC members are important factors for underpinning the crack width [19].

According to Equation (4), which is presented in the Modified Rilem model [9], the reduction coefficient of crack spacing as applied to the crack width of steel fiber can be expressed as follows:

(14) $$k=\frac{w\left(l_{f}/d_{f}+\alpha V_{f}\right)}{50\varepsilon_{sm}\left(50+0.25k_{1}k_{2}\varphi /\rho_{r}\right)}$$

## 4. Analysis of the Reduction Coefficient of Crack Spacing as Applied to Crack Width

### 4.1. Statistical Analysis of the Reduction Coefficient of Crack Spacing as Applied to Crack Width

**Figure 4:**
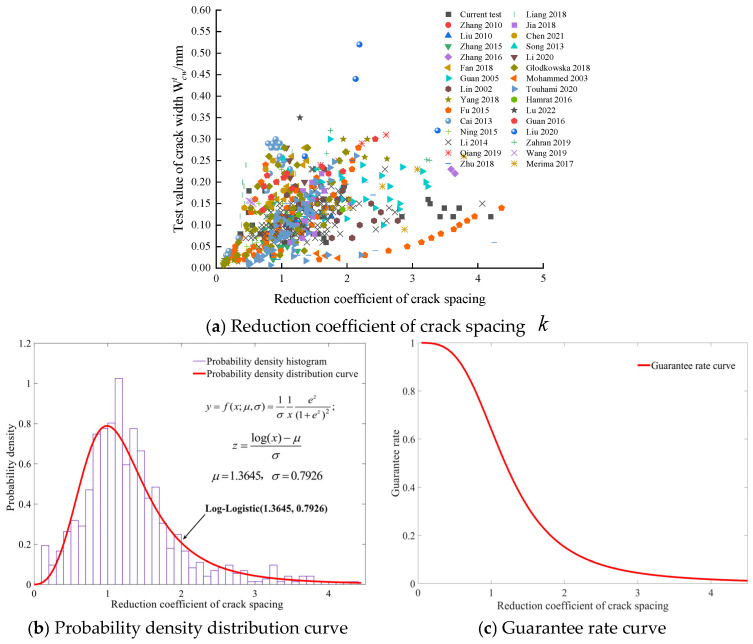
Statistical analysis of the reduction coefficient of crack spacing k [2,3,9,10,11,12,13,14,15,20,27,28,29,30,31,32,33,34,35,36,37,38,39,40,41,42,43,44,45].

Experimental data for beams with different types of steel fiber were collected, including steel fibers cut from steel wire, steel plate-shear steel fibers, and mill-cut steel fibers. Using experimental data for SFRC beams (a total of 30 groups, with 722 numerical values) [2,3,9,10,11,12,13,14,15,20,27,28,29,30,31,32,33,34,35,36,37,38,39,40,41,42,43,44,45] collected from the literature, including the concrete cover, concrete strength, reinforcement ratio, steel fiber type, and volume fraction, as well as the corresponding crack width and load results, the reduction coefficient k was ascertained through the procedures detailed in Section 3.4. The relationship between the reduction coefficient and the crack width test values is shown in Figure 4a, without considering the steel fiber type.

The test value of the reduction coefficient k varies in the range of 0.1061–4.3621. The probability density distribution curve for the test value of the reduction coefficient k is shown in Figure 4b, and it is subordinated to the Log-Logistic (1.3645, 0.7926). The area of each histogram represents the probability that k falls within the corresponding *x*-axis interval, and the total area of the histograms sums to one. The guarantee rate curve of k is shown in Figure 4c, and the ordinate value at any point on the curve indicates the probability that k exceeds the corresponding abscissa value. Figure 4c allows for the determination of k values with various guarantee rates, and shows that the guarantee rate corresponding to the reference value of 0.8 in the Modified Rilem model [9] is 78.8%.

To study the reduction coefficient k of various types of steel fiber, a comprehensive analysis was conducted on the steel fiber types and test data used in the current study and related studies. The steel fibers can be classified into three types: steel fiber cut from steel wire (a total of 14 groups, with 351 numerical values) [9,10,12,15,20,28,29,32,34,37,39,41,42], steel plate-shear steel fiber (a total of 6 groups, with 244 numerical values) [11,13,27,32,33,35], and mill-cut steel fiber (a total of 6 groups, with 99 numerical values) [30,31,32,36,40,43]; the steel fiber types for the remaining 6 groups were unspecified.

**Figure 5:**
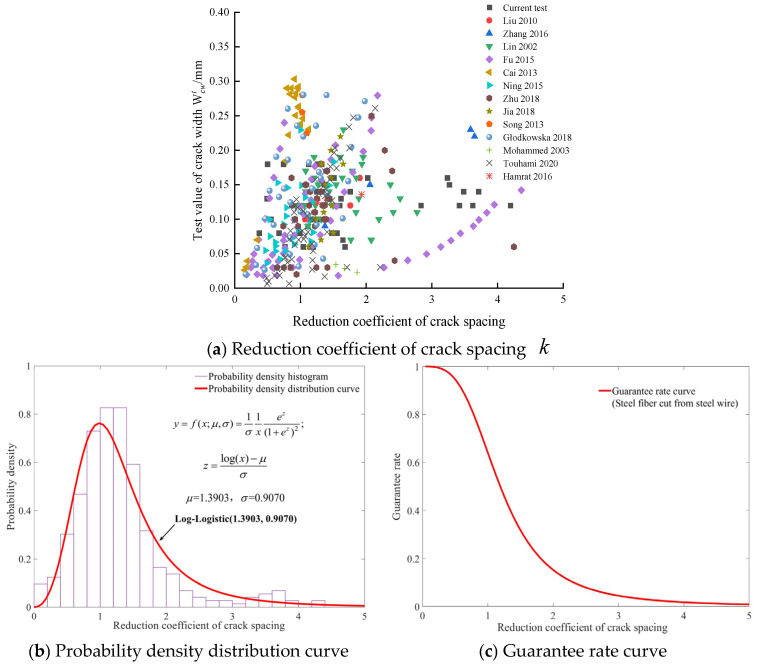
Statistical analysis of the reduction coefficient of crack spacing k for steel fiber cut from steel wire [9,10,12,15,20,28,29,32,34,37,39,41,42].

**Figure 6:**
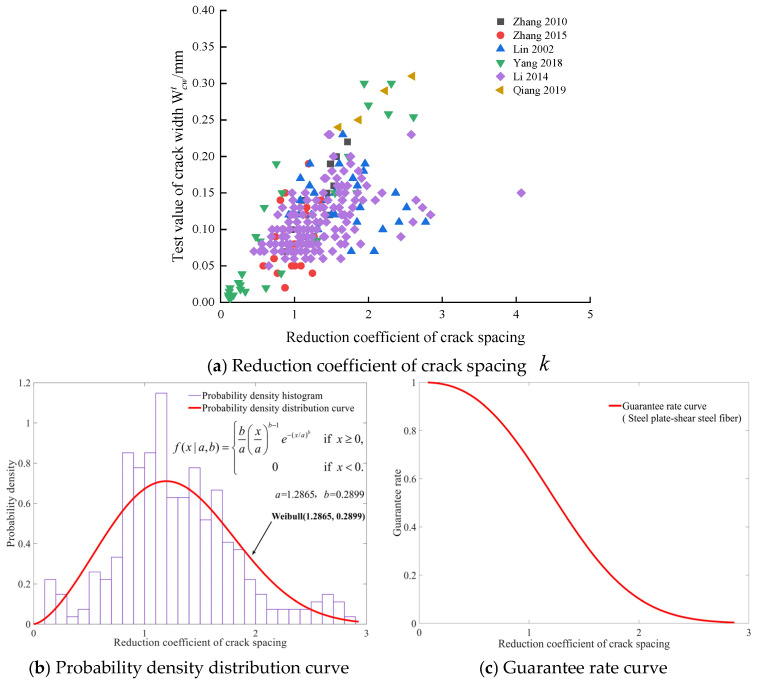
Statistical analysis of the reduction coefficient of crack spacing k for steel plate-shear steel fiber [11,13,27,32,33,35].

**Figure 7:**
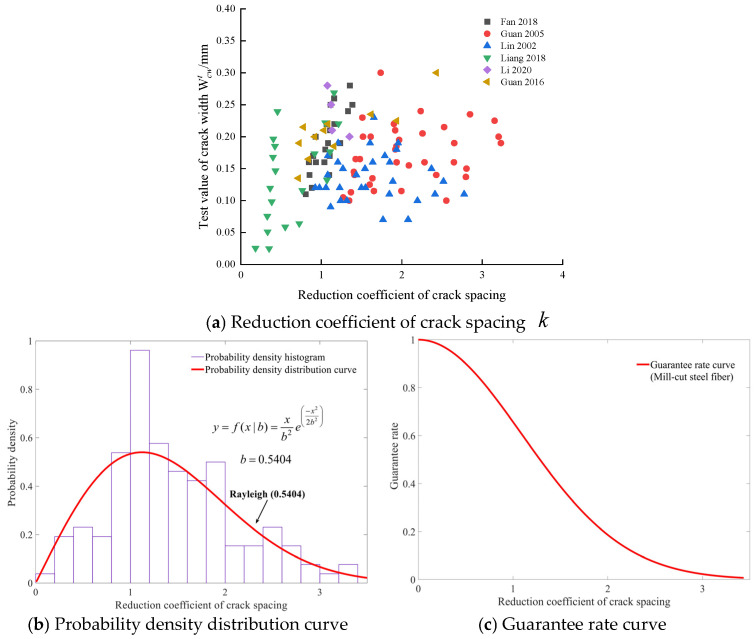
Statistical analysis of the reduction coefficient of crack spacing k for mill-cut steel fiber [30,31,32,36,40,43].

The relationship between the reduction coefficient of crack spacing k and crack width for the three types of steel fiber are shown in Figure 5a, Figure 6a, and Figure 7a. The probability density distribution curve of k for the three types of steel fiber are subject to the Log-Logistic (1.3903, 0.9070) distribution, Weibull (1.2865, 0.2899) distribution, and Rayleigh (0.5404) distribution, respectively, as shown in Figure 5b, Figure 6b, and Figure 7b. The k with different guarantee rates for various steel fiber types can be determined from Figure 5c, Figure 6c, and Figure 7c, respectively. For beams with steel fiber cut from steel wire, steel plate-shear steel fiber, and mill-cut steel fiber, the respective guarantee rates for the reduction coefficient k, corresponding to 0.8, which is recommended by the Modified Rilem model [9], are 78.29%, 80.38%, and 77.1%, respectively.

### 4.2. Suggestions for Revising the Reduction Coefficient of Crack Spacing as Applied to Crack Width

Based on the guarantee rate curves for the reduction coefficient of crack spacing k, the reduction coefficients for different steel fiber types with various guarantee rates are shown in Section 4.1. According to the Modified Rilem model [9], the crack widths were calculated when the reduction coefficients for the steel fiber were selected as 0.48, 0.6, 0.71, 0.77, and 1.19 (Figure 4c), with guarantee rates of 95%, 90%, 85%, 80%, and 50%, respectively. The calculated crack widths of 18 beam specimens and experimental results under different load conditions are compared and summarized in Table 4.

The predicted value is relatively conservative, as the predicted crack widths are less than those of the test value, when the reduction coefficient has a guarantee rate of 95%, 90%, 85%, 80%, and 50%, respectively. The predicted crack widths for the reduction coefficient k, with a guarantee rate of 50%, are closest to the experimental results, as the mean value of $W_{cw}^{t}/W_{cw}^{u5}$ is 1.3023. The mean value of $W_{cw}^{t}/W_{cw}^{u5}$ is 0.9993 for the beam teams, except for the specimens with a higher reinforcement ratio, for which the predicted results are shown to be in good agreement with experimental results. The stress and strain of longitudinal tensile steel bar are less under the same load level, while reinforcement ratios were high, for beam specimens with ID B-R2.56, B-R2.86, and B-R3.22, which plays a leading role in crack control due to the small crack width, thus, the capacity of steel fiber to prevent crack propagation in concrete members cannot be fully utilized.

As previously stated, the characteristics of 30 groups of SFRC beams, including cover thickness, reinforcement ratio, concrete strength, steel fiber type and volume fraction, crack width, and corresponding load test results, were collected from the relevant literature. The guarantee rates corresponding to the reduction coefficient of 0.8 recommended by the Modified Rilem model [9] are 78.8%, 78.29%, 80.38%, and 77.1%, respectively, for the steel fiber regardless of fiber type, the steel fiber cut from steel wire, the steel plate-shear steel fiber, and the mill-cut steel fiber. It can be seen that the reduction coefficient of 0.8 is recommended by the Modified Rilem model [9] was suitable for calculating the maximum crack width of RC beams mixed with three types of steel fiber. When considering the influence of high-strength steel bar and high-strength SFRC, the reduction coefficient for the SFRC beams was proposed to be modified as 1.19 with a guarantee rate of 50% according to statistical analysis results in this study. There are many factors affecting the crack width and crack measurement. The question of how to comprehensively factor in the variability of the steel fiber type, steel fiber volume fraction, layer thickness ratio, longitudinal reinforcement ratio, concrete strength, and section size, as well as crack width standardization measurement method, and the uncertainty of calculation mode as applied to crack width for SFRC beams, and the reduction coefficient used to calculate the crack width of SFRC beams under the appropriate guarantee rate all require further study.

## 5. Conclusions

In order to investigate the effect of steel fiber on the maximum crack width of SFRC members under normal service conditions, a series of 19 flexural beams were tested, and the applicability of the reduction coefficient to the flexural crack width of steel fiber in SFRC beams according to the Modified Rilem model for calculating maximum crack width was discussed. The reduction coefficient was used to determine the influence of steel fiber on crack width. The probability distribution and the values for different steel fiber types with various guarantee rates of the reduction coefficient were obtained, which can be used to revise the value of the reduction coefficient in the Modified Rilem model. Drawing on both the experimental results and an analytical investigation, the following conclusions were reached:

(1) The crack width decreases with the addition of steel fiber. The maximum crack width of an SFRC beam specimen with 1.5% steel fiber volume fraction reduces by more than 40% relative to the RC beam specimen under the same load.
(2) The probability density distribution curve of the reduction coefficient of crack spacing k of steel fiber is subject to the Log-Logistic (1.3645, 0.7926) distribution irrespective of steel fiber type, and to the Log-Logistic (1.3903, 0.9070) distribution, Weibull (1.2865, 0.2899) distribution, and Rayleigh (0.5404) distribution for steel fiber cut from steel wire, steel plate-shear steel fiber, and mill-cut steel fiber, respectively. The probability density distribution curve can be used to obtain the guarantee rate curve, and the guarantee rate curve provides the reduction coefficient values with different guarantee rates.
(3) The predicted crack widths are closest to the experimental results when the reduction coefficient of crack spacing k with a guarantee rate of 50%. The effect of steel fiber on inhibiting the propagation and widening of cracks can be truly reflected, thereby reducing the reduction coefficient k in Modified Rilem model can be taken as 1.19, with a guarantee rate of 50%.

## Figures and Tables

**Table 1:** Physical and mechanical properties of steel bars.

Type	Diameter (mm)	Yield Strength (MPa)	Ultimate Strength (MPa)
HPB300	10	330	503
HRB400	16	469	560
HRB500	8	559	724
HRB500	12	571	700
HRB500	14	573	760
HRB500	16	554	688
HRB500	18	548	713
HRB500	20	580	717
HRB600	16	642	833

**Table 2:** Parameters of tested beam specimens.

Specimen ID	C	H	F (%)	S (1)	R (%)	W (mm)	D (mm)	λ (1)
B-H400	CF60	HRB400	1.0	1	1.02	153	300	3.44
B-C40	CF40	HRB500	1.0	1	1.02	153	300	3.44
B-F0	C60	HRB500	0	0	1.02	150	300	3.44
B-F05	CF60	HRB500	0.5	1	1.02	154	304	3.38
B-C6H5F1S1R1.02	CF60	HRB500	1.0	1	1.02	155	305	3.37
B-F15	CF60	HRB500	1.5	1	1.02	152	305	3.37
B-C80	CF80	HRB500	1.0	1	1.02	150	308	3.33
B-H600	CF60	HRB600	1.0	1	1.02	154	305	3.37
B-F1(4D)	CF60	HRB500	1.0	1	1.02	154	305	3.37
B-F1(5D)	CF60	HRB500	1.0	1	1.02	153	304	3.38
B-R0.25	CF60	HRB500	1.0	1	0.25	152	305	3.32
B-R0.57	CF60	HRB500	1.0	1	0.57	155	303	3.37
B-R0.78	CF60	HRB500	1.0	1	0.78	154	303	3.38
B-R2.56	CF60	HRB500	1.0	1	2.56	150	300	3.79
B-R2.86	CF60	HRB500	1.0	1	2.86	152	305	3.72
B-R3.22	CF60	HRB500	1.0	1	3.22	155	300	3.82
B-S03	CF60	HRB500	1.0	0.3	1.02	150	305	3.37
B-S05	CF60	HRB500	1.0	0.5	1.02	150	305	3.37
B-S07	CF60	HRB500	1.0	0.7	1.02	149	303	3.40

Note: C is concrete strength; H is steel bar strength; F is steel fiber volume fraction; S is SFRC depth ratio in tensile zone of beam, which is calculated by dividing the thickness of SFRC at the bottom of beam by the beam height; R is longitudinal tensile reinforcement ratio; W is measured beam width; D is measured beam height; λ is shear span ratio of beam.

**Table 3:** Comparison of crack widths predicted by various codes with measured results.

Specimen ID	$P_{u}/kN$	P/kN	$w_{t}$ (mm)	$w_{p}$ (mm)							$w_{t}/w_{p}$						
				Rilem TC	Modified-Rilem	Eurocode 2	ACI 318 (Gergely & Lutz)	ACI 318 (Frosch)	GB50010 -2010	JGJ/T 465-2019	Rilem TC	Modified-Rilem	Eurocode 2	ACI 318 (Gergely & Lutz)	ACI 318 (Frosch)	GB50010-2010	JGJ/T 465-2019
B-F0	127.37	40.06	0.10	/	/	0.0401	0.1509	0.6443	0.0471	0.0471	/	/	2.4950	0.6628	0.1552	2.1242	2.1242
B-F0	127.37	60.34	0.14	/	/	0.0684	0.2272	0.6516	0.1238	0.1238	/	/	2.0473	0.6161	0.2149	1.1305	1.1305
B-F05	136.46	60.34	0.12	0.0751	0.0601	0.0662	0.2272	0.6516	0.1238	0.1097	1.5970	1.9964	1.8128	0.5281	0.1842	0.9692	1.0936
B-F05	136.46	81.52	0.16	0.1058	0.0846	0.0959	0.3070	0.6623	0.2040	0.1808	1.5124	1.8906	1.6682	0.5212	0.2416	0.7844	0.8851
B-C6H5 F1S1R1.02	148.45	58.64	0.10	0.0801	0.0640	0.0645	0.2209	0.6509	0.1174	0.0907	1.2490	1.5615	1.5500	0.4528	0.1536	0.8516	1.1024
B-C6H5 F1S1R1.02	148.45	80.12	0.15	0.1101	0.0881	0.0950	0.3017	0.6615	0.1987	0.1535	1.3624	1.7032	1.5790	0.4972	0.2268	0.7550	0.9773
B-F15	152.94	58.74	0.08	0.0808	0.0646	0.0632	0.2212	0.6509	0.1178	0.0776	0.9903	1.2381	1.2661	0.3616	0.1229	0.6792	1.0310
B-F15	152.94	79.32	0.10	0.1091	0.0873	0.0923	0.2987	0.6610	0.1957	0.1289	0.9165	1.1458	1.0837	0.3347	0.1513	0.5110	0.7757
B-F15	152.94	100.70	0.12	0.1385	0.1108	0.1225	0.3792	0.6745	0.2766	0.1822	0.8661	1.0829	0.9797	0.3164	0.1779	0.4338	0.6585
Mean											1.2134	1.5169	1.6091	0.4768	0.1809	0.9154	1.0865
SD											0.2722	0.3402	0.4492	0.1149	0.0374	0.4724	0.3964
COV											0.2243	0.2243	0.2792	0.2409	0.2068	0.5161	0.3649

Note: $P_{u}$ is the ultimate load; P is the load that corresponds to the crack width; $w_{t}$ is the measured value of the crack width; $w_{p}$ is the predicted value of crack width; Mean is the mean value; SD is the standard deviation; COV is the coefficient of variation.

**Table 4:** Comparison of crack widths predicted by the Modified Rilem model with various reduction coefficients of crack spacing with measured results.

Specimen ID	$f_{cu}/MPa$	P/KN	$W_{cw}^{t}/mm$	$W_{cw}^{u}/mm$	$W_{cw}^{t}/W_{cw}^{u}$	k	$W_{cw}^{u1}/mm$	$W_{cw}^{t}/W_{cw}^{u1}$	$W_{cw}^{u2}/mm$	$W_{cw}^{t}/W_{cw}^{u2}$	$W_{cw}^{u3}/mm$	$W_{cw}^{t}/W_{cw}^{u3}$	$W_{cw}^{u4}/mm$	$W_{cw}^{t}/W_{cw}^{u4}$	$W_{cw}^{u5}/mm$	$W_{cw}^{t}/W_{cw}^{u5}$
B-H400	61.67	38.56	0.06	0.0401	1.4949	1.1959	0.0241	2.4914	0.0301	1.9931	0.0356	1.6843	0.0386	1.5531	0.0597	1.0049
B-H400	61.67	60.54	0.1	0.0643	1.5552	1.2442	0.0386	2.592	0.0482	2.0736	0.0571	1.7524	0.0619	1.6158	0.0956	1.0455
B-H400	61.67	81.62	0.16	0.0872	1.8345	1.4676	0.0523	3.0574	0.0654	2.4459	0.0774	2.067	0.0839	1.9059	0.1297	1.2333
B-C40	48.03	39.36	0.06	0.0403	1.4907	1.1925	0.0242	2.4845	0.0302	1.9876	0.0357	1.6796	0.0387	1.5488	0.0599	1.0021
B-C40	48.03	60.04	0.1	0.0626	1.5981	1.2785	0.0375	2.6635	0.0469	2.1308	0.0555	1.8007	0.0602	1.6604	0.0931	1.0743
B-C40	48.03	81.02	0.16	0.085	1.8828	1.5063	0.051	3.138	0.0637	2.5104	0.0754	2.1215	0.0818	1.9562	0.1264	1.2658
B-F05	65.67	60.34	0.12	0.0601	1.9964	1.5971	0.0361	3.3273	0.0451	2.6618	0.0533	2.2494	0.0579	2.0741	0.0894	1.3421
B-F05	65.67	81.52	0.16	0.0846	1.8906	1.5125	0.0508	3.1509	0.0635	2.5208	0.0751	2.1302	0.0815	1.9642	0.1259	1.271
B-C6H5F1S1 R1.02	68.50	58.64	0.1	0.064	1.5615	1.2492	0.0384	2.6025	0.048	2.082	0.0568	1.7594	0.0616	1.6223	0.0953	1.0497
B-C6H5F1S1 R1.02	68.50	80.12	0.15	0.0881	1.7032	1.3625	0.0528	2.8386	0.0661	2.2709	0.0782	1.9191	0.0848	1.7695	0.131	1.145
B-F15	71.62	58.74	0.08	0.0646	1.2381	0.9905	0.0388	2.0635	0.0485	1.6508	0.0573	1.3951	0.0622	1.2863	0.0961	0.8323
B-F15	71.62	79.32	0.1	0.0873	1.1458	0.9166	0.0524	1.9096	0.0655	1.5277	0.0775	1.291	0.084	1.1904	0.1298	0.7703
B-F15	71.62	100.7	0.12	0.1108	1.0829	0.8663	0.0665	1.8048	0.0831	1.4438	0.0984	1.2201	0.1067	1.125	0.1648	0.728
B-C80	84.67	62.91	0.1	0.0689	1.4505	1.1604	0.0414	2.4175	0.0517	1.934	0.0612	1.6344	0.0664	1.507	0.1025	0.9751
B-C80	84.67	76.47	0.14	0.0841	1.6642	1.3314	0.0505	2.7737	0.0631	2.2189	0.0747	1.8751	0.081	1.729	0.1251	1.1188
B-C80	84.67	103.19	0.18	0.1139	1.5797	1.2637	0.0684	2.6328	0.0855	2.1062	0.1011	1.7799	0.1097	1.6412	0.1695	1.062
B-H600	63.35	60.44	0.13	0.0643	2.0219	1.6175	0.0386	3.3698	0.0482	2.6959	0.0571	2.2782	0.0619	2.1007	0.0956	1.3593
B-H600	63.35	81.32	0.16	0.087	1.8383	1.4707	0.0522	3.0639	0.0653	2.4511	0.0772	2.0713	0.0838	1.9099	0.1295	1.2358
B-H600	63.35	101.4	0.18	0.1088	1.6541	1.3233	0.0653	2.7569	0.0816	2.2055	0.0966	1.8638	0.1047	1.7186	0.1619	1.112
B-F1(4D)	67.60	39.16	0.08	0.0411	1.9451	1.5561	0.0247	3.2418	0.0308	2.5935	0.0365	2.1917	0.0396	2.0209	0.0612	1.3076
B-F1(4D)	67.60	59.54	0.14	0.0637	2.1977	1.7582	0.0382	3.6629	0.0478	2.9303	0.0565	2.4763	0.0613	2.2834	0.0948	1.4775
B-F1(4D)	67.60	80.72	0.16	0.0869	1.841	1.4728	0.0521	3.0683	0.0652	2.4546	0.0771	2.0743	0.0837	1.9127	0.1293	1.2376
B-F1(4D)	69.71	39.66	0.06	0.0417	1.4374	1.1499	0.025	2.3957	0.0313	1.9166	0.037	1.6196	0.0402	1.4934	0.0621	0.9663
B-F1(5D)	69.71	60.34	0.09	0.0647	1.3919	1.1135	0.0388	2.3198	0.0485	1.8558	0.0574	1.5683	0.0622	1.4461	0.0962	0.9357
B-F1(5D)	69.71	80.62	0.14	0.0869	1.6109	1.2887	0.0521	2.6848	0.0652	2.1478	0.0771	1.815	0.0837	1.6736	0.1293	1.0829
B-R0.25	66.93	25.07	0.08	0.1713	0.4671	0.3736	0.1028	0.7784	0.1285	0.6227	0.152	0.5263	0.1649	0.4853	0.2548	0.314
B-R0.25	66.93	30.17	0.13	0.2115	0.6147	0.4918	0.1269	1.0245	0.1586	0.8196	0.1877	0.6926	0.2036	0.6386	0.3146	0.4132
B-R0.25	66.93	40.06	0.18	0.2877	0.6256	0.5004	0.1726	1.0426	0.2158	0.8341	0.2554	0.7049	0.277	0.6499	0.428	0.4205
B-R0.57	65.78	40.06	0.08	0.0948	0.8435	0.6748	0.0569	1.4058	0.0711	1.1247	0.0842	0.9504	0.0913	0.8764	0.1411	0.5671
B-R0.57	65.78	60.14	0.1	0.1448	0.6908	0.5527	0.0869	1.1514	0.1086	0.9211	0.1285	0.7784	0.1393	0.7177	0.2153	0.4644
B-R0.57	65.78	80.02	0.18	0.1937	0.9293	0.7434	0.1162	1.5488	0.1453	1.239	0.1719	1.0471	0.1864	0.9655	0.2881	0.6247
B-R0.78	66.49	40.56	0.06	0.0626	0.9589	0.7671	0.0375	1.5982	0.0469	1.2786	0.0555	1.0805	0.0602	0.9963	0.0931	0.6447
B-R0.78	66.49	60.04	0.12	0.0941	1.2752	1.0202	0.0565	2.1253	0.0706	1.7003	0.0835	1.4368	0.0906	1.3249	0.14	0.8573
B-R0.78	66.49	81.22	0.18	0.1281	1.4056	1.1245	0.0768	2.3426	0.096	1.8741	0.1137	1.5838	0.1233	1.4604	0.1905	0.9449
B-R2.56	64.13	102	0.06	0.0286	2.0991	1.6793	0.0172	3.4984	0.0214	2.7988	0.0254	2.3651	0.0275	2.1809	0.0425	1.4111
B-R2.56	64.13	120.58	0.12	0.0339	3.5438	2.8351	0.0203	5.9064	0.0254	4.7251	0.0301	3.993	0.0326	3.6819	0.0504	2.3824
B-R2.56	64.13	140.66	0.16	0.0396	4.045	3.236	0.0237	6.7416	0.0297	5.3933	0.0351	4.5577	0.0381	4.2026	0.0588	2.7193
B-R2.86	65.91	122.78	0.12	0.0281	4.2742	3.4194	0.0168	7.1237	0.0211	5.699	0.0249	4.816	0.027	4.4408	0.0418	2.8734
B-R2.86	65.91	140.36	0.14	0.0321	4.3559	3.4847	0.0193	7.2598	0.0241	5.8078	0.0285	4.908	0.0309	4.5256	0.0478	2.9283
B-R2.86	65.91	160.24	0.15	0.0367	4.0837	3.2669	0.022	6.8061	0.0275	5.4449	0.0326	4.6013	0.0354	4.2428	0.0546	2.7453
B-R3.22	64.38	122.08	0.12	0.0229	5.2445	4.1956	0.0137	8.7408	0.0172	6.9926	0.0203	5.9093	0.022	5.4488	0.034	3.5257
B-R3.22	64.38	141.16	0.12	0.0265	4.5275	3.622	0.0159	7.5459	0.0199	6.0367	0.0235	5.1014	0.0255	4.7039	0.0394	3.0437
B-R3.22	64.38	160.54	0.14	0.0302	4.6389	3.7111	0.0181	7.7316	0.0226	6.1852	0.0268	5.227	0.029	4.8197	0.0449	3.1186
B-S03	62.30	27.47	0.03	0.028	1.0697	0.8557	0.0168	1.7828	0.021	1.4262	0.0249	1.2053	0.027	1.1113	0.0417	0.7191
B-S03	62.30	38.16	0.06	0.0403	1.4904	1.1924	0.0242	2.4841	0.0302	1.9873	0.0357	1.6794	0.0387	1.5485	0.0599	1.002
B-S03	62.30	58.76	0.16	0.0632	2.5306	2.0245	0.0379	4.2177	0.0474	3.3741	0.0561	2.8514	0.0609	2.6292	0.094	1.7013
B-S05	62.11	38.37	0.06	0.0409	1.4682	1.1746	0.0245	2.447	0.0306	1.9576	0.0363	1.6543	0.0393	1.5254	0.0608	0.987
B-S05	62.11	58.42	0.12	0.0634	1.8922	1.5137	0.0381	3.1536	0.0476	2.5229	0.0563	2.132	0.061	1.9659	0.0943	1.272
B-S05	62.11	78.28	0.14	0.0855	1.6371	1.3097	0.0513	2.7285	0.0641	2.1828	0.0759	1.8446	0.0823	1.7009	0.1272	1.1006
B-S07	60.87	40.86	0.08	0.0432	1.8514	1.4812	0.0259	3.0857	0.0324	2.4686	0.0383	2.0861	0.0416	1.9236	0.0643	1.2447
B-S07	60.87	61.84	0.1	0.0665	1.5037	1.203	0.0399	2.5062	0.0499	2.0049	0.059	1.6943	0.064	1.5623	0.0989	1.0109
B-S07	60.87	83.02	0.14	0.0898	1.5592	1.2474	0.0539	2.5986	0.0673	2.0789	0.0797	1.7568	0.0864	1.6199	0.1336	1.0482
Mean					1.9372	1.5497		3.2286		2.5829		2.1827		2.0126		1.3023
SD					1.1283	0.9027		1.8805		1.5044		1.2713		1.1723		0.7585
COV					0.5825	0.5825		0.5825		0.5825		0.5825		0.5825		0.5825

Note: $f_{cu}$ is the cubic compressive strength of concrete; p is load; $W_{cw}^{t}$ is measured crack width under the corresponding load; k is the reduction coefficient of crack spacing; $W_{cw}^{u}$, $W_{cw}^{u1}$, $W_{cw}^{u2}$, $W_{cw}^{u3}$, $W_{cw}^{u4}$, and $W_{cw}^{u5}$ are the predicted crack widths, with k selected as 0.8, 0.48, 0.6, 0.71, 0.77, and 1.19, respectively, where 0.8 was recommended by Modified Rilem model, and others were determined according to the results of statistical analysis, with a guarantee rate of 95%, 90%, 85%, 80%, and 50%, respectively; Mean is the mean value; SD is the standard deviation; COV is the coefficient of variation.

## Data Availability

The original contributions presented in this study are included in the article. Further inquiries can be directed to the corresponding author.
